# Living with axial spondyloarthritis: a cross-sectional survey of patient knowledge and perceptions

**DOI:** 10.1007/s00296-024-05637-x

**Published:** 2024-06-22

**Authors:** Olena Zimba, Zofia Guła, Magdalena Strach, Mariusz Korkosz

**Affiliations:** 1 Department of Rheumatology, Immunology and Internal Medicine, University Hospital in Kraków, Kraków, Poland; 2 National Institute of Geriatrics, Rheumatology and Rehabilitation, Warsaw, Poland; 3 Department of Internal Medicine N2, Danylo Halytsky Lviv National Medical University, Lviv, Ukraine; 4 Department of Rheumatology and Immunology, Jagiellonian University Medical College, Kraków, Poland

**Keywords:** Axial spondyloarthritis, Physicial activity, Lifestyle, Diagnostic delays, Treatment, Rehabilitation, Surveys and questionnaires

## Abstract

Diagnosis and effective treatment of axial spondyloarthritis (AxSpA) are often delayed due to inadequate awareness and poor patient-physician communication. Some AxSpA patients fail to maintain an active lifestyle by exercising regularly, further worsening their disease management. The evolving concept of patient-centred care necessitates better understanding of patient awareness and their needs. We aimed to survey AxSpA patients to reflect on healthcare planning and management perspectives. Our self-administered questionnaire focused on perceptions of AxSpA diagnosis and management, particularly exploring issues of physical activity and active lifestyle. Satisfaction with AxSpA medical care and its accessibility, diagnostic delays, patient-physician communication, and support for disease management were also explored. This offline survey was arranged at the Department of Rheumatology, Immunology, and Internal Medicine of Jagiellonian University Medical College and Krakow University Hospital. We surveyed patients with AxSpA attending outpatient clinics between December 1st, 2023 and April 22nd, 2024. The questionnaire included questions on types of physical activities, barriers to exercising, satisfaction with medical care, patient-physician interactions, diagnostic delays, and use of teleconsultations. A total of 117 patients with AxSpA were enrolled (mean age 41.62 years). The majority (*n* = 93, 79.5%) were employed. There was a male predominance (69, 59%). The average diagnostic delay was 5.5 years. Notably, 104 (88.9%) responders perceived physical activity as a factor influencing their disease course. However, only 32 (27.35%) managed to exercise regularly (≥ 30 min, 2–3 times a week). The majority (70, 59.83%) were irregularly engaged in some form of physical activity, with 15 (12.8%) not exercising at all, and nearly half (48%) reported at least one barrier to maintaining a physically active lifestyle. Pain (32, 27.35%), fatigue (27, 23.08%), lack of motivation (17, 14.53%), and lack of time (12, 10.26%) were noted as barriers to exercising. The respondents preferred to exercise at home. The survey identified critical areas where patient dissatisfaction or uncertainty were notably prevalent: 38 (32.5%) were uncertain and 35 (30%) were dissatisfied with rehabilitation access. For spa therapy, 63 (53.85%) reported uncertainty and 23 (19.7%) expressed dissatisfaction. Only 48 (41%) were treated by a rehabilitation specialist last year. Only 23% of AxSpA patients took part in teleconsultations last year, and 65% preferred in-person visits. While AxSpA patients recognize the importance of physical activity, significant barriers exist to engaging them regularly in exercising. Addressing these barriers through personalized, motivational, and educational strategies could improve patient outcomes. Improving patient satisfaction with healthcare services, particularly in areas of rehabilitation and physician-patient communication, is crucial for improving the overall care of AxSpA patients.

**Supplementary Information:**

The online version contains supplementary material available at 10.1007/s00296-024-05637-x.

## Introduction

Axial spondyloarthritis (AxSpA) is a chronic inflammatory disease primarily targeting the spine and sacroiliac joints [1]. The diagnosis of AxSpA and its effective treatment are often delayed due to patients’ and health professionals’ lack of understanding of the disease and their poor communication [2]. This gap in understanding and communication adversely affects the diagnostic workflow and delays the treatment initiation, worsening patient well-being [3]. The resultant challenges in maintaining an active lifestyle and engaging in regular physical activities further complicate the management of AxSpA [4].

The evolving concept of AxSpA management that emphasizes patient-centered care necessitates better understanding of patient perspectives on their disease and unmet needs. An improved understanding of patients’ knowledge and perceptions is crucial for optimizing education efforts and tailoring management strategies to patient needs [5].

Despite the proven benefits of regular physical activities for AxSpA patients, misperceptions and related challenges hinder their engagement in such activities. One of the primary barriers to physical activities is the fluctuating nature of AxSpA symptoms, with periods of exacerbations severely affecting patient motivation and mobility [6]. The lack of tailored exercise schemes for AxSpA patients with diverse symptoms further contributes to the uncertainty of opting for the most effective and safe exercise therapies [7]. The quantitative approach to exercise therapies for alleviating pain, improving mobility, and establishing disease control remains elusive [8]. The scarcity of specialized physical therapy resources, particularly in regions with poor healthcare infrastructure and where the distance from the service facility is often remote and long waiting times for community rehabilitation services, may further confound the situation. Patient fears and misconceptions about exercise exacerbating AxSpA symptoms may necessitate tailored education schemes and professional support.

Our research employed a survey-based approach to garner AxSpA patient opinion essential for improved healthcare planning and management. This study focused on bridging the literature gap regarding patient perspectives on AxSpA diagnosis and management, particularly exploring the unmet needs in expanding physical activities and improving health management.

This survey aimed to uncover the prevailing exercising trends and barriers that AxSpA patients face while attempting to maintain a physically active lifestyle. The frequency, type, and intensity of exercise were explored in this survey. By surveying a group of Polish patients, we also aimed to explore patient satisfaction with AxSpA medical care in Poland and reflect on healthcare accessibility, diagnostic delays, patient-physician communication, and overall support for disease management. Patient satisfaction is particularly important in the context of treatment adherence, disease outcomes, and quality of life. By examining confounders of Polish AxSpA patient satisfaction, such as availability of treatments, quality of patient-physician communication, and support for disease management, we attempted to identify the key areas for improving patient-centered care.

## Patients and methods

This survey was arranged at the Department of Rheumatology, Immunology, and Internal Medicine of Jagiellonian University Medical College and Krakow University Hospital. It aligned with the objectives of the “POLNOR-RHEUMA” project, which is dedicated to enhancing the healthcare quality and improving the health outcomes of patients with rheumatic diseases in Poland [9].

### Participants

The participants were consecutive patients diagnosed with AxSpA recruited at the outpatient clinic between 1 December 2023 and 22 April 2024. The eligibility criteria included confirmed diagnosis of AxSpA according to the latest applicable classification criteria [10], age above 18 years, and ability to give informed consent. Patients with cognitive impairments were excluded.

### Survey design and validation

The survey questionnaire was designed to capture insights into patient experience with AxSpA, including aspects related to diagnosis, management, physical activity, and satisfaction with medical care (Appendix 1). The questionnaire development involved an extensive review of current literature, practice guidelines, outcomes from large cohort studies, and findings from previous AxSpA surveys. We adhered to previously published recommendations on survey designing, conducting, and reporting [11].

The survey aimed to evaluate AxSpA patients’ knowledge, perceptions, and management of their disease, with an emphasis on physical activity and satisfaction with medical care. The questionnaire covered a range of topics, from diagnostic awareness to detailed inquiries about exercise habits, smoking status, comorbidities, and medicamentous and non-medicamentous therapies. Participants were asked about their satisfaction levels with various aspects of rheumatological care in Poland, including access to rheumatologists, information about the disease, treatment discussions, and treatment effectiveness.

The survey questions were structured to capture types of physical activities, barriers to exercising, and preferences for managing disease exacerbation. The survey also included points on teleconsultations, therapies under the National Health Fund’s targeted treatment reimbursement programme, and concerns about medication side effects. Demographic data, including age, gender, nationality, education level, and employment status, were also collected. The questionnaire validation was arranged by expert review to ensure clarity, relevance, and comprehensiveness of the questions. The survey was pretested with a small subset of the target population to refine the questions and revise the questionnaire based on the patient feedback.

### Survey administration

The survey was administered in paper format to facilitate patients’ completion during clinic visits. Participation was voluntary and anonymous, without disclosure of identifiable personal information to ensure confidentiality. Patients were asked to return their completed surveys to a deposit ‘ballot’ box in reception. The anonymous nature of the survey was aimed to enhance the reliability and validity of the collected data. The survey methodology was designed to adhere to the highest ethical standards, with approval by the Institutional Review Board (IRB) of the Jagiellonian University Medical College (protocol N 118.6120.07.2023, June 15, 2023). All participants provided informed consent before completing the questionnaire, with the assurance that their responses would be used solely for research purposes and would not affect their medical care.

### Statistics

Convenience sampling was employed to recruit real-world AxSpA patients attending outpatient clinics. We employed descriptive statistics to report frequencies. Graphics were drawn using Microsoft Excel (Microsoft, Redmond, WA, USA).

## Results

A total of 117 patients with AxSpA were enrolled (mean ± SD age 41.62 ± 11.54 years). The demographic data indicate a diverse group consisting of Polish subjects with diverse educational backgrounds. The majority (93, 79.5%) were employed, and there was a considerable male predominance (69, 59%). Hypertension was identified as the most common comorbidity (30, 25.6%), followed by osteoarthritis (21, 17.9%). Other comorbidities included Hashimoto thyroiditis and inflammatory bowel disease (each present in 5.12% of the surveyed population), and fibromyalgia (3.4%).

### Diagnostic delay

The diagnostic delay, the average time from the onset of disease symptoms to the diagnosis by rheumatologist, was 5.5 years.

### Awareness of disease subtypes

The data revealed a significant gap in awareness about subtypes of AxSpA, with nearly half of the respondents (49, 42%) were unaware of the distinction between radiographic and non-radiographic forms of AxSpA. While 33 (28.2%) understood this distinction, a considerable number (35, 29.9%) remained unsure, indicating a need for enhanced educational efforts to improve the understanding of AxSpA subtypes.

### Physical activity patterns

#### Distribution of regular versus irregular exercisers

**Fig. 1:**
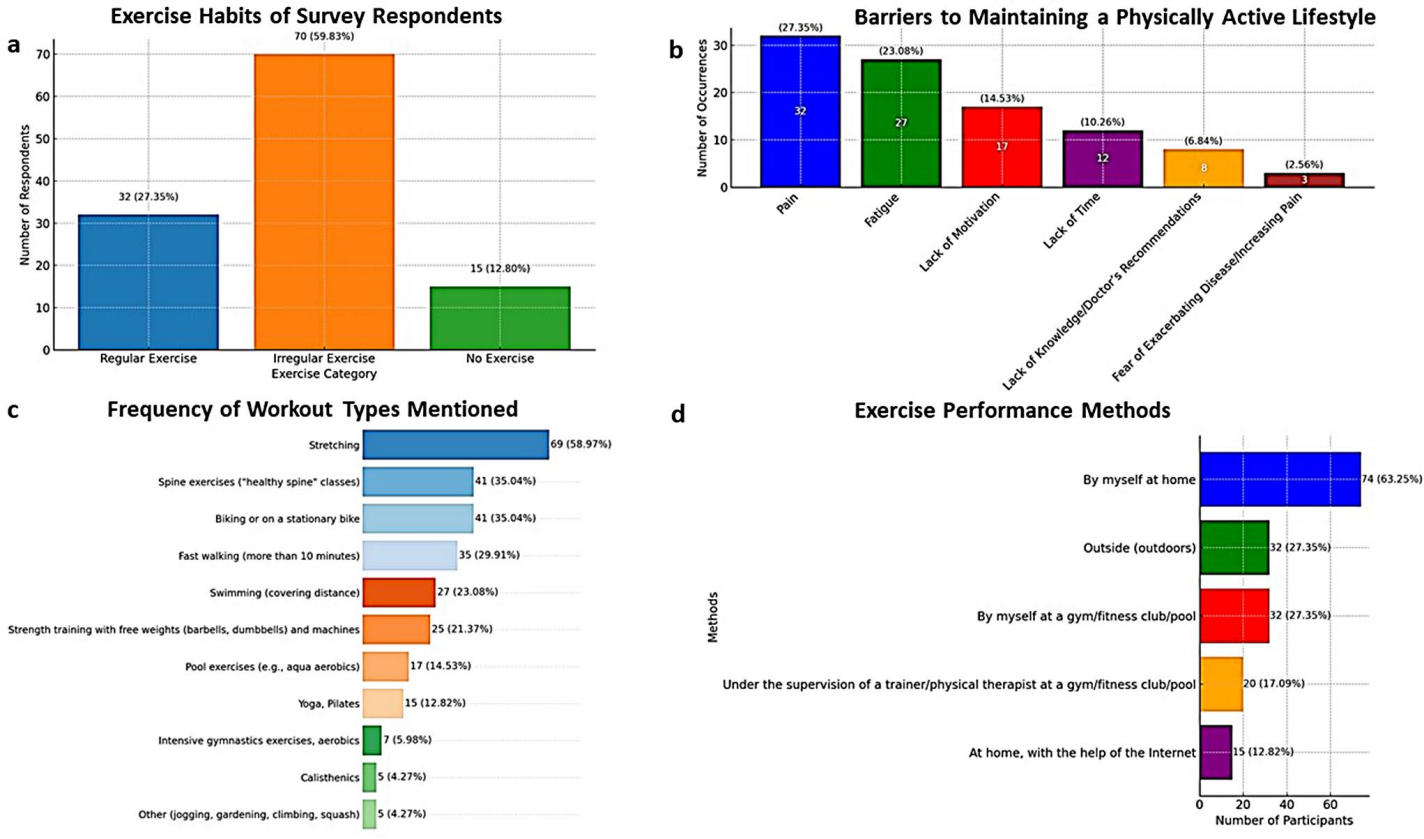
Analysis of physical activity patterns among AxSpA patients: exercise habits (**a**), barriers (**b**), workout types (**c**), and exercise performance methods (**d**)

Remarkably, 104 (88.9%) responders highlighted physical activity as a factor influencing their disease course, suggesting a unanimous perception of its importance. However, only 32 (27.35%) managed to exercise regularly (≥ 30 min, 2–3 times a week or more). The majority (70, 59.8%) were engaged in some form of physical activity, though irregularly, with 15 (12.8%) were not exercising at all (Fig. 1a).

#### Barriers to maintaining a physically active lifestyle

Out of 117 participants, 56 (47.9%) reported at least one barrier to maintaining a physically active lifestyle (Fig. 1b). Pain was identified as a primary deterrent to exercise by 32 (27.35%), 27 (23.1%) reported fatigue as one of the most significant barriers, and 17 (14.53%) mentioned lack of motivation to exercise. Notably, 12 (10.3%) stated that lack of time prevents them from exercising. A smaller number of respondents indicated that lack of knowledge or doctor’s recommendations on how to exercise correctly (8, 6.84%) and fear of exacerbating the disease or increasing pain (3, 2.6%) were barriers to their engagement in physical activities.

#### Types of workouts and exercise performance methods

The types of workouts diagram depicts a range of physical activities, pointing to solo activities and stretching as the most popular, followed by spine exercises and biking (Fig. 1c). The exercise performance methods diagram presents the respondents’ exercise preferences, such as exercising at home, followed by outdoor and gym activities (Fig. 1d). The majority of patients (93, 79.5%) preferred to exercise alone, while a smaller group (9, 7.7%) opted for group activities, and 15 (12.8%) did not engage in any form of exercise. The preference to exercise alone may reflect the need for convenience, comfort, and personal space. Biking, swimming, and fast walking were also popular, suggesting a preference for aerobic exercises adjustable to individual fitness levels.

#### Smoking

Although nearly half of the respondents (53, 45.3%) recognized smoking as a factor influencing their disease and well-being, many patients continued to smoke. In fact, 20 (17.1%) were current tobacco smokers, and 4 (3.4%) were e-cigarette users. A smaller percentage of respondents reported using both tobacco and e-cigarettes (2, 1.7%). This scenario highlights the gap between disease awareness and lifestyle choices, underscoring the importance of not just educating patients about the risks associated with smoking, including the progression of structural damage, but also supporting them in smoking cessation efforts.

#### Patient satisfaction with different aspects of rheumatology care

**Fig. 2:**
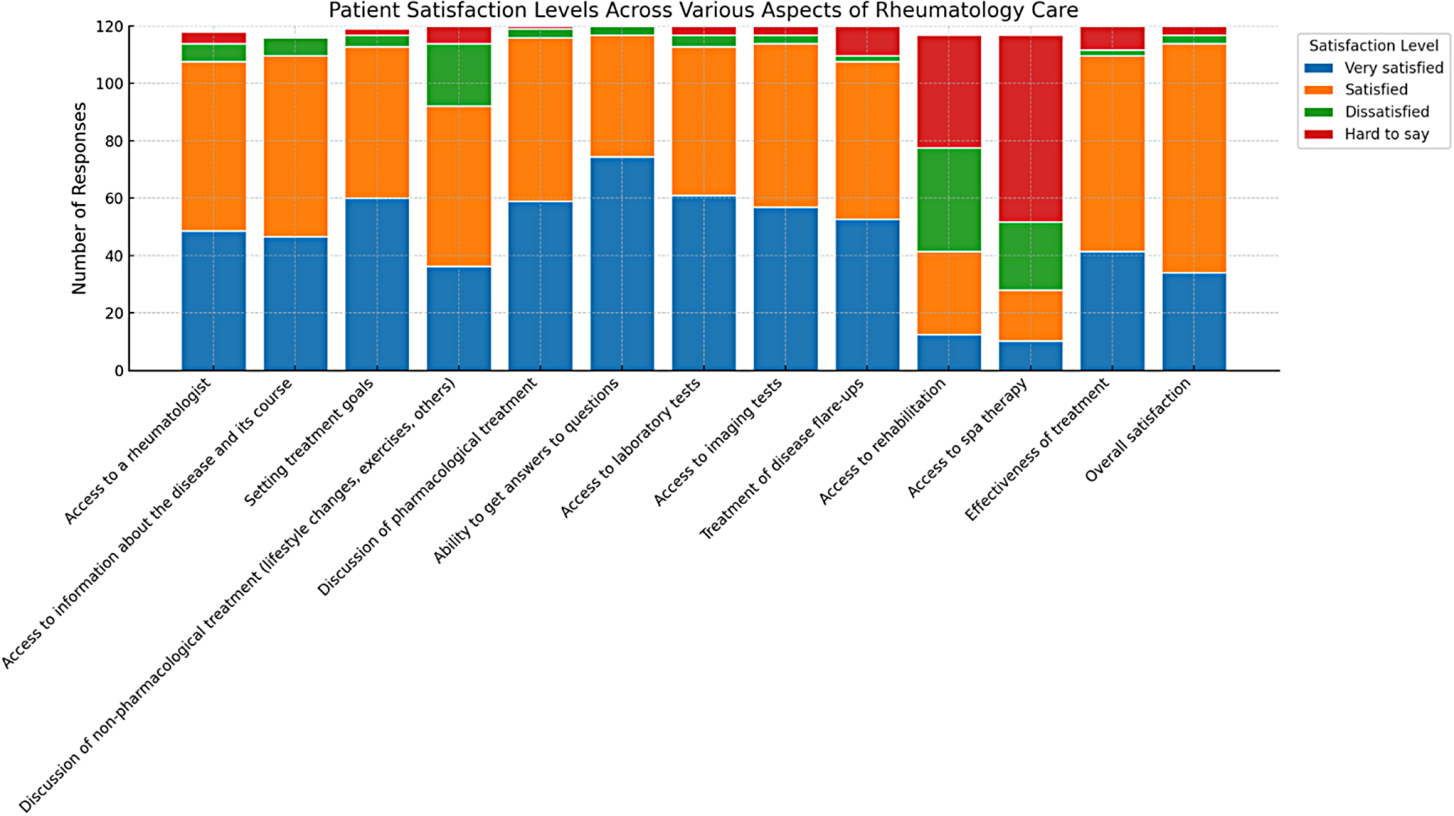
The distribution of patient satisfaction levels across various aspects of rheumatology care

Figure 2 illustrates the distribution of patient satisfaction related to different aspects of rheumatology care in Poland. Each bar in Fig. 2 represents a different aspect of medical care, with segments indicating the number of responses for each satisfaction level: “Very (Extremely) Dissatisfied,” “Dissatisfied,” “Satisfied,” “Very (Extremely) Satisfied,” and “Hard to Say.”

The overwhelming majority of patients were satisfied (106, 90.6%) with their treatment effectiveness. The overall satisfaction also scored remarkably high at 94%. This trend extends to accessibility and quality of diagnostic services. Access to laboratory tests was reported as satisfactory by 103 (88%) respondents. Access to imaging tests showed a similarly positive response, with 108 (92.3%) patients reporting satisfaction levels from ‘Satisfied’ to ‘Very (Extremely) Satisfied’.

#### Areas with notable dissatisfaction or uncertainty

This survey identified critical areas where patient dissatisfaction or uncertainty was prevalent: 38 (32.5%) were uncertain, and 35 (29.9%) were dissatisfied with their access to rehabilitation services. For spa therapy, 63 (53.85%) reported uncertainty and 23 (19.7%) expressed dissatisfaction. Approximately one-fourth of the feedback indicated dissatisfaction or ambiguity about the treatment of disease flare-ups and non-pharmacological treatments. These data underscore the need for targeted service improvements to enhance patient satisfaction and overall care quality in rheumatology practices.

#### Patient access and demand for physiotherapy

Interestingly, 48 (41%) were examined or treated by physiotherapist or rehabilitation specialist and 67 (57.3%) did not receive physiotherapy in the last 12 months. The majority (67.5%) expressed a desire to receive a referral to physiotherapist or rehabilitation specialist. In contrast, only 17.95% of respondents were not interested in receiving the referral, and 14.53% remained undecided. This disparity between the low incidence of treatment and expressed interest in referrals highlights a gap in service provision and accessibility to rehabilitation.

#### Suggestions regarding better rheumatology care

Only 55 (47%) indicated satisfaction with current management practices, stating that “Nothing needs to be changed.” However, there remains a demand for enhanced patient-physician communication and patient education: 38 (32.5%) emphasized the importance of discussing the disease course with their doctor and receiving tailored advice. Twenty-nine (24.8%) highlighted the need to regularly monitor spine and sacroiliac joint mobility, advocating for more rigorous and routine physical assessments. The results also revealed a desire for more frequent follow-up visits (7, 6%), reflecting a call for increased interaction with healthcare providers.

#### Perception of AxSpA exacerbations

A significant number of respondents (47, 40.2%) proactively contacted their rheumatologist *via* email, SMS, or phone, while 29 (24.8%) called their rheumatology clinic registration to arrange an appointment. Twenty-seven (23.1%) patients preferred to consult their family doctor as an initial step. Notably, 12 (10.25%) managed exacerbations by increasing their NSAID dose. Beyond medical consultations, 4.24% of patients were engaged in exercises and an equal percentage seeked advice through social media or Google, indicating a reliance on both physical activity and online resources for managing symptoms. These insights point to diverse approaches to self-management and care-seeking behaviour among patients with AxSpA.

#### Teleconsultation use and preference

**Fig. 3:**
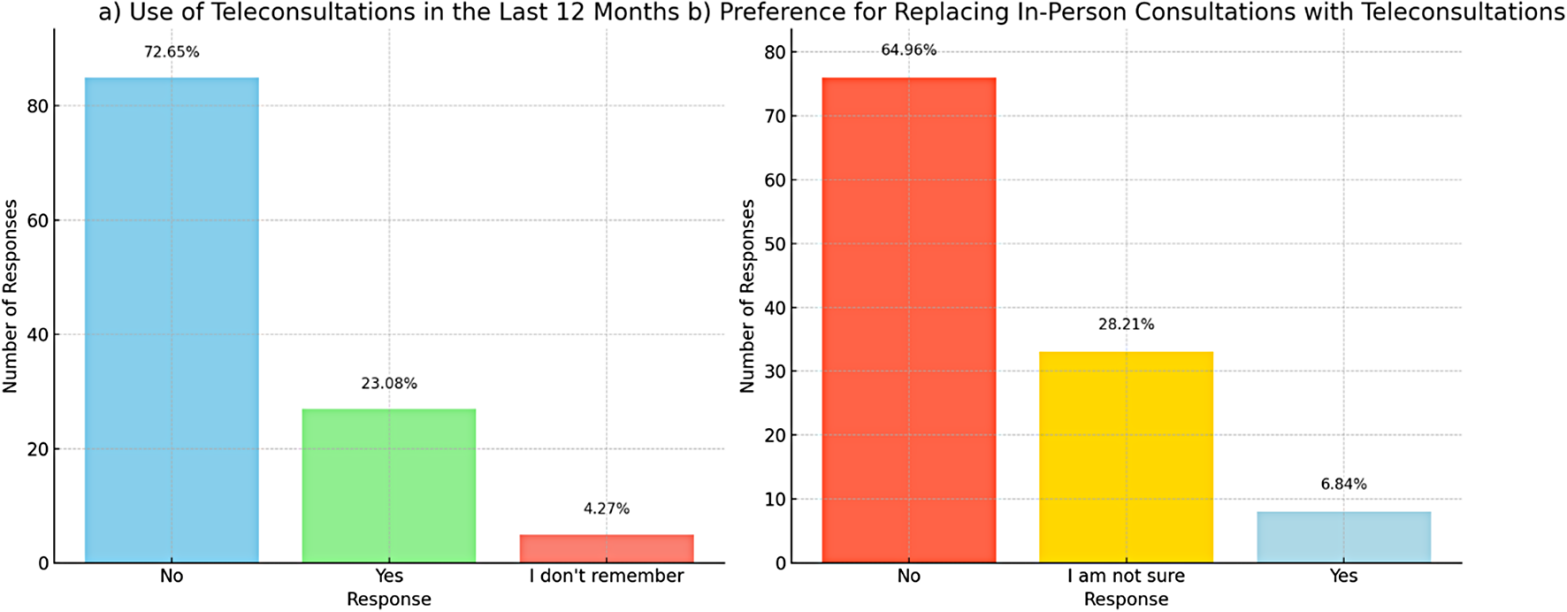
The use of teleconsultations among patients with AxSpA in the last 12 months and their preferences for replacing in-person consultations with teleconsultations

Only 27 (23.1%) utilized teleconsultations with rheumatologist in the last year (Fig. 3a). The majority (85, 72.65%) did not take part in teleconsultations. When asked about the prospect of replacing in-person consultations with teleconsultations, the majority (76, 65%) preferred to maintain in-person visits and only 8 (6.8%) supported the idea of switching to teleconsultations (Fig. 3b). Additionally, 33 (28.2%) were still uncertain about this substitution. Despite the growing availability of telehealth services, there remains a strong preference for traditional face-to-face interactions, possibly reflecting the nuanced and highly personalized nature of AxSpA care.

#### Concerns regarding treatment side effects

TNF inhibitors were frequently used (61, 52.1%), followed by IL-17 inhibitors (32, 27.35%). Only a small subset were treated with JAK inhibitors (2, 1.7%). NSAIDs were widely used by responders (64, 54.7%). A variety of other analgesic medications were utilized: paracetamol (30, 25.6%), metamizole (6, 5.1%), and weak opioid analgesics such as tramadol (5, 4.3%). A smaller subset of patients also reported using anticonvulsant medications such as pregabalin and gabapentin (1.7%). A small number of patients reported using oral steroids (7, 6%). Concerns about side effects were significant, with 37 (31.6%) expressing apprehension regarding their current drug therapies, highlighting the need for continuous monitoring and patient education to mitigate these concerns.

## Discussion

The current survey offers insights into AxSpA patients’ knowledge and satisfaction with medical care. We revealed AxSpA diagnostic delay of 5.5 years, with potentially adverse effects on patient outcomes. This delay is shorter than that reported by the broader European International Map of Axial Spondyloarthritis (IMAS) survey (7.4 years), where Poland was not represented [3], and by the US version of the IMAS survey (8.8 years) [12]. These differences in country-based diagnostic delays may be indicative of the efficiency of diverse healthcare management and patient education strategies. The inherent limitation of diagnostic delay studies and reported variations can be associated with self-reported data, implying recall bias and uncertainties with recording AxSpA symptoms’ onset. A systematic review on diagnostic delays in AxSpA proved that substantial delays are still reported despite significant advances in diagnostic procedures and disease awareness campaigns [13]. The global diagnostic delays range from 2 to 6 years [2].

Our analysis sheds light on AxSpA patients’ physical activity patterns. Although regular physical activity is crucial for reducing pain and stiffness, improving range of motion, and increasing muscle strength, with additive effects on drug therapies [4, 8], only one-third of our patients exercise regularly. Most respondents were engaged in physical activities irregularly or did not exercise at all. Moderate-intensity aerobic exercise for at least 30 min five days a week is advised at all stages of AxSpA [6].  Overall, aerobic exercises are known to reduce disease activity and improve physical function in AxSpA patients [14]. The latest systematic review indicates that aquatic exercise effectively manages pain, disease activity, and physical function in AxSpA patients [15]. Exercise guidelines incorporate postural education which can improve morning stiffness, spinal mobility, thoracic expansion, and overall quality of life [6]. A Cochrane systematic review indicates that individual home-based or supervised exercise programmes are superior to no intervention [16]. Additionally, supervised exercise therapies have beneficial effects on fatigue, mood, and general health of AxSpA patients [17].

In view of the importance of physical activity in managing AxSpA and revealed challenges with patients’ engagement in regular exercises, one can conclude that there is a huge gap between patient knowledge and action. This gap is in line with the results of previous studies of patients’ awareness of exercise benefits and barriers to enforcing related actions [18].

Our findings reveal numerous barriers to physical activity, with pain, fatigue, lack of motivation, and time constraints being predominant. These barriers are not unique to Polish AxSpA patients since the same challenges have been reported globally [19]. The fear of exacerbating symptoms while exercising is particularly concerning since it points to a lack of patient confidence in exercising safely. The fear or avoidance behaviour suggests that catastrophizing movement-related pain leads to a fear of movement and activity avoidance, worsening disability [20].

In spite of numerous barriers and challenges, a subset of our respondents managed to engage in regular or irregular physical activities. Presumably, interventions aimed at managing pain and fatigue, enhancing motivation, and addressing time management might increase physical activity among our respondents. By incorporating regular physical activity into the treatment plan, healthcare professionals can help improve the overall well-being of their AxSpA patients. Personalised exercise programmes may enhance long-term adherence by considering patient preferences, capabilities, and current health status assessments, including musculoskeletal, psychosocial, and AxSpA-specific measures such as axial mobility and chest expansion [8]. Integrating motivational interviewing techniques into patient consultations may help to overcome motivational barriers and enhance exercise participation [21]. Exercise therapy is safe for most patients, but it should be tailored to the individual needs of those with severe and advanced AxSpA due to risks associated with bony changes, balance and mobility issues, osteoporosis, and cardiorespiratory consequences [8]. Movements should be limited to comfortable ranges to prevent complications. Complete immobilization should be avoided during painful periods since staying active alleviates pain and enhances spinal mobility [6]. Supervising patients through online communication and follow-ups is advisable. Individual feedback is crucial to build confidence and proficiency in physical exercise [6].

Nearly half of our patients acknowledged the harmful impact of smoking on their health, though many continued to smoke. This gap highlights the need for more effective interventions that extend beyond merely communicating the risk of smoking. Comprehensive support is required to incorporate cessation therapies, counselling, and encouragement from healthcare providers to facilitate smoking cessation. There is a link between tobacco smoking and spondyloarthritis activity [22]. History of smoking is dose-dependently associated with more severe disease phenotype in AxSpA patients [23].

We revealed a dissonance between patient satisfaction with diagnostic services and dissatisfaction with access to reimbursed rehabilitation and spa therapies. A gap exists in physiotherapy demand and actual access: 67.5% of responders supported referrals, while only 41% received the desired treatment. The obtained data underscore an unmet need, possibly due to limitations in service availability imposed by the national payers. Addressing these challenges requires strategic country-based health planning with increased funding, workforce expansion, cost-effectiveness confirmation and efforts to enhance patient education on the availability and advantages of much-desired therapies.

Approximately one-fourth of our patients reported dissatisfaction or uncertainty regarding discussions of non-pharmacological treatments. These patients requested improved communication to gain a thorough understanding of the options, benefits, and implementation of these treatments. Enhanced education programmes, offering detailed information through materials or workshops, and tailored treatment discussions could cater to individual patient needs and preferences. Furthermore, establishing mechanisms for patient feedback could help identify and address barriers to implementing these treatments.

Additionally, 38 (32.5%) respondents stressed the importance of discussing their disease course and receiving personalized advice. Addressing related issues may mitigate their dissatisfaction with current treatment discussions, but it requires a rearrangement of the time devoted to the patient by the doctor and other healthcare workers.

Our survey reports a range of self-management strategies during exacerbations. Notably, 47 (40%) of our patients directly contacted their rheumatologist, 29 (25%) scheduled appointments through their clinic registration, and 27 (23%) consulted their family doctor, showcasing a strong preference for professional medical advice. Additionally, 12 (10.25%) adjusted their NSAID therapies, and 8.5% relied on exercises and online resources. These behaviours align with literature findings that emphasize a high level of patient initiative in chronic disease management [24]. Overall, these data underscore the importance of patient education for effective self-management in AxSpA.

There is a relatively low uptake of teleconsultations among our surveyed patients (23%). Among those who had teleconsultations, there was a clear tendency to opt for in-person consultations. In view of reported diagnostic delays, telemedicine may shorten these delays by improving access to care. Studies on telehealth in rheumatology focus on video and phone consultations [25]. The asynchronous physician-based telemedicine approach to diagnose AxSpA with access to imaging results may be helpful for correct diagnosis [26].

The COVID-19 pandemic catalyzed a shift in healthcare delivery, transitioning from in-person to telehealth consultations [27]. This change presented varied experiences: while some patients experienced a seamless transition, others faced technical challenges and struggled with the new interaction mode [28]. Concerns about the quality of care emerged, particularly regarding the impersonality of digital interactions and the inability to perform physical examinations, leading some to question the overall effectiveness of telehealth [29].

Disparities in access to digital technologies posed significant barriers. Despite these challenges, the COVID-19 pandemic fostered a broader acceptance of telehealth and recognized its convenience and safety [30]. A better understanding of related patient experiences is crucial for refining telehealth strategies and addressing concerns to enhance telehealth services post-pandemic.

### Implications for practice and research

Healthcare providers should customize exercise advice and support according to the individual needs of AxSpA patients. Effective communication about all treatment aspects, including reimbursed rehabilitation and spa therapies, is also critically important. From a research perspective, there is a need to investigate strategies that effectively bridge the gap between patient knowledge and behaviour, particularly concerning exercising. Future research should focus on evaluating various exercise interventions, motivational techniques, and educational programmes to enhance exercise adherence. Longitudinal studies can help track changes in patient knowledge, perceptions, and behaviours, offering insights into the effectiveness of interventions and the evolution of patient experiences with AxSpA management. Expanding research to include diverse patient populations may improve the generalizability of findings and reveal regional differences in patient experiences.

Studies examining the impact of digital health tools such as mobile apps and online platforms may identify new ways to improve access to information and support for patients with AxSpA.

### Study limitations

The current survey relies on self-reported data, which may be biased due to recall issues. Patients may not accurately remember past health behaviours. As a cross-sectional study, our survey captures a snapshot in time, limiting the ability to infer causality or changes over time in patient knowledge, perceptions, and behaviors regarding AxSpA management. And finally, the convenience sampling cannot represent the broader AxSpA patient population. Our participants’ geographic location, study setting (single centre), and non-randomized recruitment could influence the findings’ generalizability.

## Conclusion

While AxSpA patients recognize the importance of physical activity in managing their disease, significant barriers prevent them from engaging in regular exercises that could have beneficial long-term effects on disease progression, activity and quality of life. Addressing these barriers through personalized, motivational, and educational strategies may improve a wide variety of patient outcomes. Enhancing patient satisfaction with healthcare services, particularly in areas of rehabilitation and patient-physician communication, is crucial for improving the overall care of AxSpA patients.

### Electronic supplementary material

Below is the link to the electronic supplementary material.

Supplementary Material 1

## Data Availability

The corresponding author can provide raw data upon reasonable request.
