# Supplementary material for: Living with axial spondyloarthritis: a cross-sectional survey of patient knowledge and perceptions
Source: Rheumatol Int. 2024 Jun 22;44(8):1543–52. doi: 10.1007/s00296-024-05637-x (PMC11222235; doi:10.1007/s00296-024-05637-x)
Supplement: Supplementary file 1 — Supplementary Material 1 [file 296_2024_5637_MOESM1_ESM.pdf]

Patient knowledge and perceptions of diagnosis and management of axial spondyloarthritis

The current survey is aimed to examine patients' knowledge and perceptions of axial spondyloarthritis (AxSpA) diagnosis and management. Special attention is given to patients' satisfaction with medicamentous and non-medicamentous therapies and options for rheumatology follow-up assessments. This questionnaire is designed based on previous similar questionnaires and literature reviews. The respondents are expected to completely fill this questionnaire and share their knowledge and experience as patients managed by healthcare specialists. By completing this survey questionnaires, respondents give their agreement to publicize their answers. Although there are some personal demographic questions about responders, all answers will be kept confidential and anonymised. Only questionnaires with complete answers to all questions will be processed.
Thank You for your professional cooperation on this matter!
For further questions, Associate Professor Olena Zimba can be contacted at zimbaolena@gmail.com

Please mark one or several answers in the chosen field with an "X"

1. Based on your knowledge, what is the current diagnosis made by the physician?

- ☐ Ankylosing spondylitis
- ☐ Axial spondyloarthritis
- ☐ Non-radiographic axial spondyloarthritis
- ☐ Undifferentiated spondyloarthritis
- ☐ Other

2. Do you know that axial spondyloarthritis (AxSpA) is divided into a radiographic form, known as ankylosing spondylitis, and a non-radiographic form?

- ☐ Yes
- ☐ No
- ☐ I am not sure

3. Which of the following factors, in your knowledge, influences the course of the disease?

- ☐ Physical activity
- ☐ Diet
- ☐ Smoking cigarettes
- ☐ Alcohol
- ☐ Relaxation techniques/avoiding stressful situations
- ☐ Alternative medicine

4. What is your smoking status:

- ☐ Never smoked
- ☐ Former tobacco smoker
- ☐ Current tobacco smoker
- ☐ Passive smoker (living with a smoker)
- ☐ Current e-cigarette user
- ☐ Former e-cigarette user

5. Physical activity

- ☐ I cannot exercise due to pain
- ☐ I cannot exercise due to fatigue
- ☐ I cannot exercise due to lack of time
- ☐ I cannot exercise due to lack of motivation
- ☐ I cannot exercise due to lack of knowledge/doctor's recommendations on how to exercise correctly
- ☐ I cannot exercise due to fear of exacerbating the disease/increasing pain
- ☐ I perform regular exercises (*regular ≥30 minutes 2-3 times a week or more)
- ☐ I perform irregular exercises

6. How do you perform exercises?

- ☐ By myself at home
- ☐ At home with the help of the Internet (YouTube videos, Instagram, Facebook, and other social media platforms)
- ☐ Outside (outdoors)
- ☐ By myself at a gym/fitness club/pool
- ☐ Under the supervision of a trainer/physical therapist at a gym/fitness club/pool
- ☐ I do not exercise at all

7. You most often perform exercises:

- ☐ Alone
- ☐ In a group
- ☐ I do not exercise at all

8. Types of workouts

- ☐ Stretching
- ☐ Spine exercises (e.g., "healthy spine" classes)
- ☐ Yoga
- ☐ Pilates
- ☐ Calisthenics
- ☐ Pool exercises (e.g., aqua aerobics)
- ☐ Intensive gymnastics exercises, aerobics
- ☐ Strength training with free weights (barbells, dumbbells) and machines
- ☐ Fast walking (more than 10 minutes)
- ☐ Swimming (covering distance)
- ☐ Biking or on a stationary bike
- ☐ I do not exercise at all
- ☐ Other - please specify

9. What comorbidities have you been diagnosed with?

- ☐ Diabetes
- ☐ Hypertension
- ☐ Fibromyalgia
- ☐ Osteoarthritis
- ☐ Other - please specify

1	4
2	5
3	6

10. Please provide an approximate date of the onset of disease symptoms (lower back pain, back pain, heel pain, arthritis, inflammation of the eye's vascular membrane/iris).

month of the year ____________

year- ____________

11. **Please provide an approximate date of the disease diagnosis by a rheumatologist (ankylosing spondylitis or axial spondyloarthritis).**

month of the year [ ]

year - [ ]

12. **Who first diagnosed you with AxSpA?**

- ☐ Rheumatologist
- ☐ Family doctor
- ☐ Orthopedist
- ☐ Physiotherapist
- ☐ Gastroenterologist
- ☐ Ophthalmologist
- ☐ Dermatologist
- ☐ Other specialists – please specify [ ]

13. **Who is currently managing the treatment of AxSpA?**

- ☐ Rheumatologist
- ☐ Family doctor
- ☐ Orthopedist
- ☐ Physiotherapist
- ☐ Gastroenterologist
- ☐ Ophthalmologist
- ☐ Dermatologist
- ☐ Other specialists – please specify [ ]

14. **How often does the rheumatologist examine you and assess the course of the disease?**

- ☐ More frequently than every 3 months
- ☐ Every 3 months
- ☐ Every 6 months
- ☐ Every 12 months
- ☐ Less frequently

15. **Please evaluate your satisfaction with the quality of various aspects of rheumatological care in Poland:**

	Very dissatisfied	Dissatisfied	Satisfied	Very satisfied	Hard to say
Access to a rheumatologist	☐	☐	☐	☐	☐
Access to information about the disease and its course	☐	☐	☐	☐	☐
Setting treatment goals	☐	☐	☐	☐	☐
Discussion of non-pharmacological treatment (lifestyle changes, exercises, others)	☐	☐	☐	☐	☐
Discussion of pharmacological treatment	☐	☐	☐	☐	☐
Ability to get answers to questions	☐	☐	☐	☐	☐
Access to laboratory tests	☐	☐	☐	☐	☐
Access to imaging tests	☐	☐	☐	☐	☐
Treatment of disease flare-ups	☐	☐	☐	☐	☐
Access to rehabilitation	☐	☐	☐	☐	☐
Access to spa therapy	☐	☐	☐	☐	☐

Effectiveness of treatment	☐	☐	☐	☐	☐
Overall satisfaction	☐	☐	☐	☐	☐

16. What would you like to change in how rheumatological care is managed?

☐ Nothing needs to be changed
☐ The possibility of more frequent follow-up visits
☐ Discussing the course of the disease with the doctor and getting advice
☐ Regular monitoring of imaging tests
☐ Regular monitoring of spine and sacroiliac joint mobility
☐ Other - please specify

17. What do you usually do when you notice an exacerbation of disease symptoms?

☐ I call the rheumatology clinic to schedule an appointment (via hotline)
☐ I schedule an appointment with the family doctor
☐ I contact my rheumatologist (email, SMS, phone)
☐ I look for advice through social media/Google
☐ Other - please specify

18. Have you used teleconsultations with a rheumatologist in the last 12 months?

☐ Yes
☐ No
☐ I don't remember

19. Would you like to replace in-person consultations with teleconsultations?

☐ Yes
☐ No
☐ I am not sure

20. In the last 12 months, have you been assessed/treated by a physiotherapist/rehabilitation specialist?

☐ Yes
☐ No
☐ I am not sure

21. Would you like to receive a referral to a physiotherapist/rehabilitation specialist?

☐ Yes
☐ No
☐ I am not sure

22. Do you take non-steroidal anti-inflammatory drugs? (ibuprofen, diclofenac, naproxen, meloxicam, lornoxicam, ketoprofen, nimesulid, celecoxib, etoricoxib)

☐ Yes
☐ No
☐ I am not sure

23. Please list the pain relief medications you use

☐ Paracetamol
☐ Metamizole
☐ Non-steroidal anti-inflammatory drugs **(**ibuprofen, diclofenac, naproxen, meloxicam, lornoxicam, ketoprofen, nimesulid, celecoxib, etoricoxib)
☐ Opioid pain relievers, e.g. tramadol
☐ Anticonvulsant medications – pregabalin, gabapentin
☐ I am not sure
☐ Other - please specify

24. Do you take oral steroids?

- ☐ Yes
- ☐ No
- ☐ I am not sure

25. Have you received steroid injections (so-called "blocks") in the last 12 months?

- ☐ Yes
- ☐ No
- ☐ I am not sure

26. Are you currently undergoing treatment with the following medications (pills or injections)?

- ☐ Methotrexate
- ☐ Sulfasalazine
- ☐ Leflunomide
- ☐ None of the above
- ☐ I am not sure

27. Are you being treated under the National Health Fund's (NFZ) biological treatment program (medicine collected from the hospital pharmacy)?

- ☐ Yes
- ☐ No
- ☐ I am not sure

28. If so, what medication

- ☐ Inhibitor TNF (adalimumab, etanercept, certolizumab, golimumab, infliksymab)
- ☐ Inhibitor IL-17 (sekukinumab, ixekizumab)
- ☐ Inhibitory JAK (tofacytynib, upadactynib)
- ☐ None of the above
- ☐ I am not sure
- ☐ I do not remember

29. Do you have concerns about the side effects of the drug therapies you are using?

- ☐ Yes
- ☐ No
- ☐ I am not sure

PLEASE PROVIDE DEMOGRAPHIC DATA

30. Date of birth

Day (DD) Month (MM)

Year (YYYY)

31. What is your gender?

- ☐ Male
- ☐ Female
- ☐ I prefer not to answer

32. What is your nationality?

33. What is your highest level of education?

- ☐ Primary
- ☐ Secondary
- ☐ Higher

34. Are you currently employed?

- ☐ Yes
- ☐ No
- ☐ I prefer not to answer

35. Do you have any comments about the survey?

- ☐ Yes
- ☐ No

If Yes, please provide details:

DATE OF SURVEY COMPLETION

DD MM

YYYY
